# The evolution of thermal performance in native and invasive populations of *Mimulus guttatus*

**DOI:** 10.1002/evl3.275

**Published:** 2022-02-13

**Authors:** Aleah Querns, Rachel Wooliver, Mario Vallejo‐Marín, Seema Nayan Sheth

**Affiliations:** ^1^ Department of Plant and Microbial Biology North Carolina State University Raleigh North Carolina 27695; ^2^ Division of Biology Kansas State University Manhattan Kansas 66506; ^3^ Department of Biosystems Engineering and Soil Science University of Tennessee Knoxville Knoxville Tennessee 37996; ^4^ Biological and Environmental Sciences University of Stirling Stirling FK9 4LA United Kingdom

**Keywords:** Adaptive divergence, evolutionary ecology, invasion ecology, latitudinal gradient, niche conservatism, phenotypic cline, thermal performance curve, thermal tolerance

## Abstract

The rise of globalization has spread organisms beyond their natural range, allowing further opportunity for species to adapt to novel environments and potentially become invaders. Yet, the role of thermal niche evolution in promoting the success of invasive species remains poorly understood. Here, we use thermal performance curves (TPCs) to test hypotheses about thermal adaptation during the invasion process. First, we tested the hypothesis that if species largely conserve their thermal niche in the introduced range, invasive populations may not evolve distinct TPCs relative to native populations, against the alternative hypothesis that thermal niche and therefore TPC evolution has occurred in the invasive range. Second, we tested the hypothesis that clines of TPC parameters are shallower or absent in the invasive range, against the alternative hypothesis that with sufficient time, standing genetic variation, and temperature‐mediated selection, invasive populations would re‐establish clines found in the native range in response to temperature gradients. To test these hypotheses, we built TPCs for 18 native (United States) and 13 invasive (United Kingdom) populations of the yellow monkeyflower, *Mimulus guttatus*. We grew clones of multiple genotypes per population at six temperature regimes in growth chambers. We found that invasive populations have not evolved different thermal optima or performance breadths, providing evidence for evolutionary stasis of thermal performance between the native and invasive ranges after over 200 years post introduction. Thermal optimum increased with mean annual temperature in the native range, indicating some adaptive differentiation among native populations that was absent in the invasive range. Further, native and invasive populations did not exhibit adaptive clines in thermal performance breadth with latitude or temperature seasonality. These findings suggest that TPCs remained unaltered post invasion, and that invasion may proceed via broad thermal tolerance and establishment in already climatically suitable areas rather than rapid evolution upon introduction.

**Impact Summary**

Understanding the ecological and evolutionary processes that promote species invasions is of broad interest. One way species could become invasive is by rapidly evolving in response to climatic gradients in the introduced range. However, if the native and invasive ranges have similar climates, then there might be limited evolution upon introduction. To test this idea, we compared growth responses to temperature in invasive and native populations of yellow monkeyflower that occupy broad latitudinal and climatic gradients. We did not find evidence for rapid evolution in the invasive range, and instead found that invasive populations showed similar growth responses to temperature as native populations. Because thermal tolerances of invasive populations were as broad as those of native populations, broad thermal tolerance, rather than rapid evolution in the introduced range, likely contributed to the invasion success of this species.

Invasive species are one of the greatest threats to biodiversity, but the factors that contribute to a species becoming invasive are still debated (Sala et al. 2000; Lee 2002; van Kleunen et al. 2015; IPBES 2018). Baker's classic description of “ideal weeds” suggested that invasive species originate from “general‐purpose” genotypes with broad climatic tolerance (Baker 1965). However, it is unknown whether climatic tolerance in a species’ invasive range is preexisting, which predicts niche conservatism, or evolved, which predicts niche lability between the native and invasive range (Petitpierre et al. 2012; Atwater et al. 2018; Liu et al. 2020). Upon introduction to a new geographic region, a species may expand, contract, or maintain its climatic niche space depending on changes in selection, genetic drift, and gene flow between the native and the invasive range (Broennimann et al. 2012; Dlugosch et al. 2015). Similarly, a species’ average niche conditions (i.e., niche “position”) may or may not shift in the invasive range (Guisan et al. 2014). If climatic niches are conserved between species’ native and invasive ranges, then building climatic niche models based on native occurrences to identify areas with high invasion risk holds great promise for predicting and managing biological invasions (Thuiller et al. 2005; Chapman et al. 2017; Da Re et al. 2020). Numerous studies have used correlative niche models to assess climatic niche conservatism across species’ native and invasive ranges (Peterson et al. 2003; Kriticos et al. 2013; Da Re et al. 2020), but most have neglected intraspecific variation in physiological tolerances across climatic gradients (some exceptions include Ebeling et al. 2008; Hill et al. 2013). We use an experimental approach to evaluate the evolution of physiological tolerances within and between a species’ native and invasive ranges.

One dimension of a species’ climatic niche that influences its ability to invade new areas is temperature. Thermal performance curves (TPCs; Figure 1A) describe the performance of a genotype, individual, population, or species across a temperature gradient (Huey and Stevenson 1979), where performance represents some measure of an organism's ability to function (Angilletta 2009). Although TPCs are not strictly equivalent to thermal niches unless the performance metric is total fitness, they provide a powerful means of experimentally approximating a species’ fundamental thermal niche as physiological tolerance. Thus, comparisons of TPCs may not explicitly measure thermal niche conservatism between a species’ native and introduced ranges, but they shed light on whether the evolution of thermal tolerance plays a role in species’ abilities to invade. Two TPC parameters that reflect adaptation to temperature gradients are thermal optimum and thermal performance breadth (Huey and Stevenson 1979; Figure 1A). Thermal optimum details the temperature at which maximum performance is achieved (Huey and Stevenson 1979), analogous to thermal niche position. Comparable to thermal niche breadth, thermal performance breadth describes the span of temperatures across which a specified percentage of the maximum performance is achieved (Huey and Stevenson 1979), with generalists having wider breadth than specialists.

**Figure 1:**
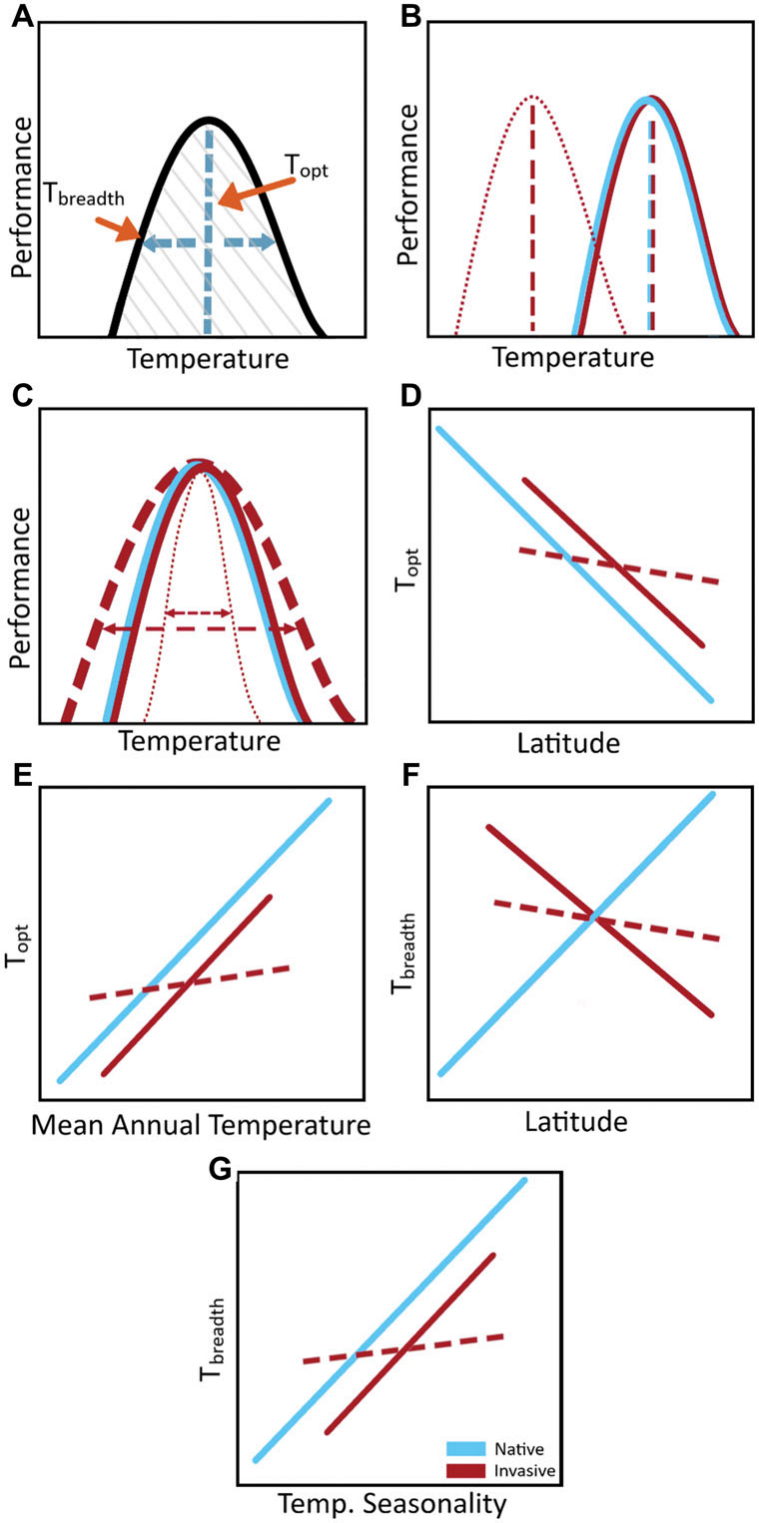
Hypotheses describing the evolution of thermal performance curves (TPCs) and clines in native (blue) and invasive (red) populations of *M. guttatus*. (A) TPC parameters of interest include thermal optimum (*T*_opt_) and thermal performance breadth (*T*_breadth_). (B) Native and invasive populations may exhibit similar *T*_opt_ (solid lines) because populations occupy similar thermal regimes or if *T*_breadth_ is maintained in the invasive range. Alternatively, invasive populations may exhibit a different *T*_opt_ relative to native populations (dotted line) due to genetic drift (Colautti et al. 2017). (C) If native and invasive thermal regimes and genetic variation in TPCs are similar, *T*_breadth_ may be maintained (solid line). Alternatively, invasive populations may evolve wider (dashed line) or narrower (dotted line) *T*_breadth_ relative to native populations due to admixture of multiple source populations (Lavergne and Molofsky 2007) or reduced temperature variation, respectively. (D and E) The native range (solid blue line) may exhibit clines such that *T*_opt_ decreases with latitude (D) and increases with mean annual temperature (E), and these clines may be re‐established (solid red) or weaker/nonexistent (dashed red) in the invasive range. (F and G) The native range may exhibit clines such that *T*_breadth_ increases with latitude (F) and temperature seasonality (G). The invasive range, where temperature seasonality increases with latitude (Figure 2D), may re‐establish clines (solid line) such that *T*_breadth_ decreases with latitude (F) and increases with temperature seasonality (G), or may exhibit weaker/no clines (dashed line).

Evolutionary divergence of TPC parameters between invasive and native populations of the same species may reflect thermal niche contraction, expansion, and/or shifts in the introduced range. If populations possess broad thermal performance breadths, and/or if temperature regimes are similar in both ranges, then native and invasive populations may exhibit similar thermal optima (Guisan et al. 2014). Alternatively, changes in thermal optima may accompany divergence in average local thermal regimes between the native and invasive range (Angilletta 2009), consistent with niche shifts. If source populations already possess plastic genotypes, they may be predisposed toward having a broad thermal niche (Ainsworth and Drake 2020), leading to similar thermal performance breadths in native and invasive populations. Alternatively, if the degree of thermal variation (e.g., temperature seasonality) differs in the native and introduced ranges, then thermal breadth could differ between invasive and native populations (Zerebecki and Sorte 2011; Bates et al. 2013), reflecting niche expansions or contractions. Further, genetic drift and admixture of source populations could result in maladaptive shifts of TPC parameters, such that thermal optimum and breadth differ in the invasive range despite similar thermal environments as the native range. Ultimately, the likelihood of thermal niche conservatism versus evolution in a species’ introduced range depends on the interplay between colonization history, genetic diversity, and temperature‐mediated selection (Wares et al. 2005; Sheth and Angert 2014; Eyster and Wolkovich 2021). To date, although there is some evidence for climatic niche conservatism during invasion, few empirical studies compare TPCs of native and invasive populations of a single species (but see Comeault et al. 2020), particularly in plants.

Given a temperature or latitudinal gradient, populations with sufficient time and genetic variation may evolve clines in TPC parameters in response to temperature‐mediated selection (Endler 1977; Diamond et al. 2017; Campbell‐Staton et al. 2018). Failure to account for the presence of latitudinal or environmental clines can mask inferences of trait evolution between native and invasive ranges, as divergent selection may occur among populations (Colautti et al. 2009). Adaptation to local thermal regimes should result in thermal optimum increasing with mean annual temperature (Angert et al. 2011), and thermal performance breadth increasing with temperature seasonality (or the “climate variability hypothesis”; Dobzhansky 1950; Janzen 1967; Stevens 1989; Gutiérrez‐Pesquera et al. 2016). If invasive populations lack sufficient time or standing genetic variation for adaptation to novel temperature gradients, or if temperature‐mediated selection in the introduced range is weak, then phenotypic clines may be shallower in the invasive range than in the native range, or absent altogether (Bhattarai et al. 2017). Alternatively, the re‐establishment of phenotypic clines across latitudinal or climatic gradients in invasive ranges would indicate that rapid evolution plays a role in the invasion process (Huey et al. 2000; Hernández et al. 2019; van Boheemen et al. 2019). However, even populations in the native range may not exhibit strong clines if they consist of generalist genotypes.

In this study, we compare TPCs of invasive and native perennial populations of the yellow monkeyflower, *Mimulus guttatus* (Phrymaceae; Figure 2A,B), also known as *Erythranthe guttata* (Fraga 2018; Lowry et al. 2019), to investigate the extent to which invasive populations conserve the thermal niche of native populations and re‐establish phenotypic clines across climatic gradients. *Mimulus guttatus* is an herbaceous plant native to western North America, occupying wet habitats across a broad latitudinal and climatic gradient from Alaska to Northern Mexico (Fraga 2018). In 1812, *M. guttatus* was brought to the United Kingdom for horticultural use and has subsequently become widespread across the British Isles and to a lesser extent parts of continental Europe (Preston et al. 2002; Truscott et al. 2008; Newman 2015). Genomic data suggest that genotypes of *M. guttatus* in the United Kingdom originated from perennial populations in Alaska (Puzey and Vallejo‐Marín 2014; Pantoja et al. 2017), followed by multiple introductions of perennial populations from across the native range (Vallejo‐Marín et al. 2021). The invasive U.K. range thus consists of a highly admixed melting pot of native populations and is not likely constrained by low genetic diversity (Puzey and Vallejo‐Marín 2014; Pantoja et al. 2017; Vallejo‐Marín et al. 2021). U.K. populations are considered locally dominant invaders and served as a bridgehead for establishment worldwide (Vallejo‐Marín et al. 2021). Climatic niche models indicate niche conservatism between North American, U.K., and European populations of *M. guttatus*, although invasive populations occur in a subset of conditions occupied in the native range (Da Re et al. 2020). The introduction of *M. guttatus* to continental Europe via U.K. populations constituted multiple introduction events (Tokarska‐Guzik and Dajdok 2021), which could have given rise to newly divergent genotypes and phenotypes. These continental European populations are closely related but distinct from U.K. populations, and even further divergent from native relatives due to secondary admixture (Vallejo‐Marín et al. 2021). Due to their well‐characterized origins, genomic composition, and pivotal role in subsequent invasions of continental Europe, New Zealand, and eastern North America (Vallejo‐Marin et al. 2021), invasive U.K. populations of M. guttatus are an obvious first choice for evaluating conservatism of thermal performance during invasion.

**Figure 2:**
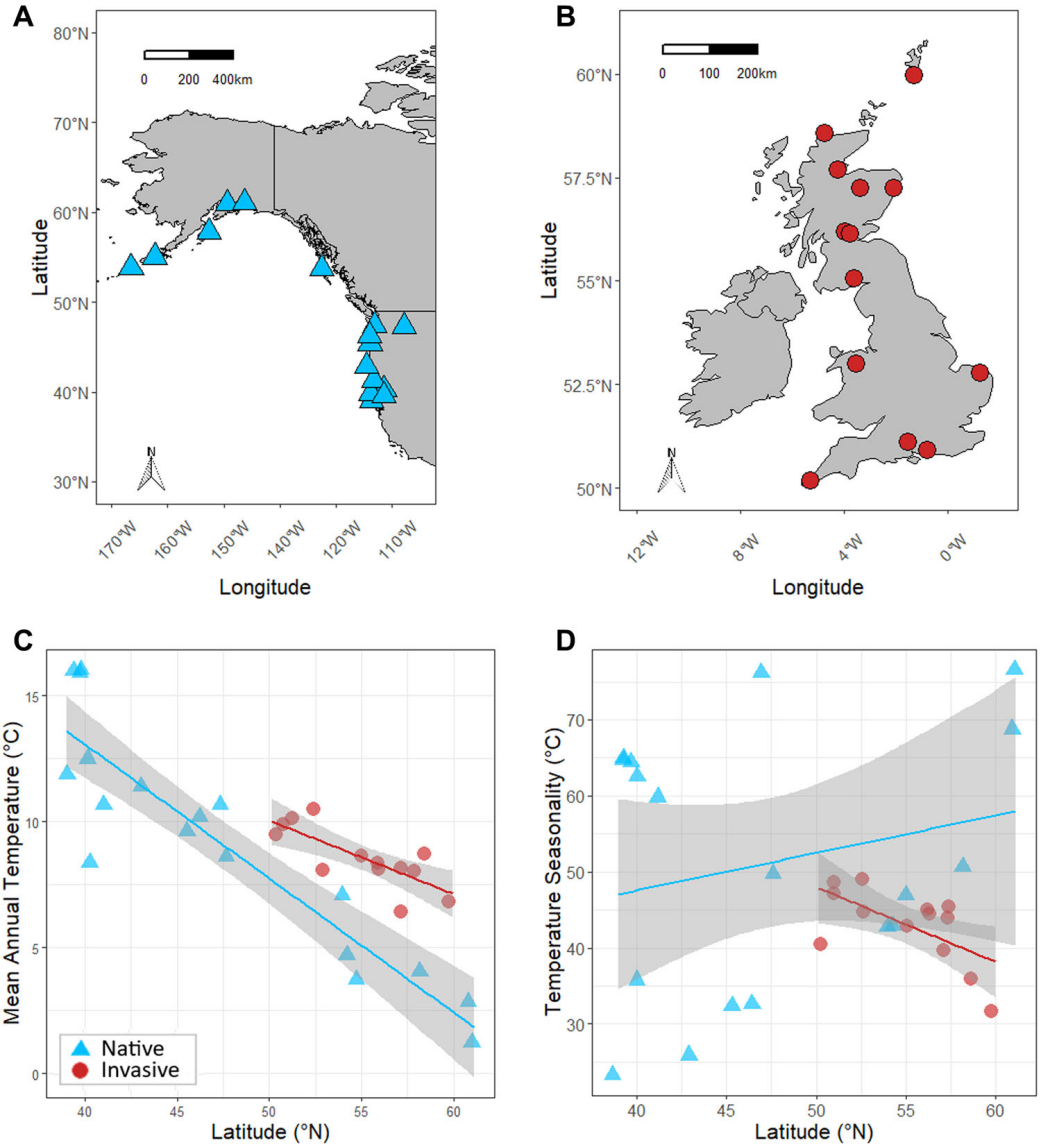
Map of focal populations of *M. guttatus* in (A) the native range in North America and (B) the invasive range in the United Kingdom. Relationships between (C) mean annual temperature and (D) temperature seasonality with latitude for native and invasive populations. Fitted lines indicate predicted mean annual temperature or seasonality as a function of latitude based on linear models. The gray shaded area represents a 95% confidence interval for these predictions. A minor jitter effect has been added to (C) and (D) to improve visibility of points. Climate data were obtained from WorldClim version 2 (∼1‐km resolution, 1970–2000, Fick and Hijmans 2017).

Here, we assess hypotheses about thermal niche conservatism versus evolution during the U.K. invasion of *M. guttatus*. First, due to similar thermal regimes in the native and invasive ranges (Figure 2C,D), we hypothesized that thermal performance parameters would not differ between ranges (Figure 1B,C), consistent with thermal niche conservatism. Alternatively, if the species has undergone significant changes in genetic variation via genetic drift and/or admixture in its invasive range, there may be differences in thermal optima and performance breadths between the native and invasive ranges (Figure 1B,C). Second, we hypothesized that adaptive clines of TPC parameters are shallower or absent in the invasive range (Figure 1D–G) due to weak selection across a narrow thermal gradient (Figure 2C,D). Alternatively, invasive populations could re‐establish clines of thermal performance parameters found in the native range through rapid adaptation to temperature gradients in the invasive range (Figure 1D–G). Specifically, thermal optimum should increase with mean annual temperature (Figure 1E), which decreases with latitude in both ranges (Figures 1D and 2C), and thermal performance breadth should increase with temperature seasonality (Figure 1G), which increases with latitude in the native range but decreases with latitude in the invasive range (Figures 1F and 2D).

## Materials and Methods

### PLANT PROPAGATION

In June 2019, we grew seeds from 18 North American and 13 U.K. populations of perennial *M. guttatus* (Figure 2A,B; Table S1) in the North Carolina State University Phytotron. Although some of the coastal populations in our study have been renamed *Erythranthe grandis* (Nesom 2012), many researchers continue to call them “*Mimulus guttatus*” because they are interfertile with other members of the species (Lowry and Willis 2010). We used an average of three seed families from populations spanning a range of latitudes selected to capture most of the occupied thermal gradient in the native and invasive ranges, totaling 95 unique genotypes (Table S1). These plants, kept in large pots (8‐inch diameter, 7‐inch depth) in a growth room with a 20/15°C day/night temperature regime, served as the source of replicate cuttings for thermal performance experiments and remained in the growth room for the duration of the experiment. Eight weeks after the initial planting, we cut the primary stem of each plant to its base to encourage branching, which is ideal for taking replicate cuttings. See *Methods* in the Supporting Information for further details on population selection and plant propagation.

### THERMAL PERFORMANCE EXPERIMENT

Ten weeks after planting, plants possessed sufficient vegetative growth to support many cuttings. To generate thermal performance data for each genotype within each population, we took cuttings of similar sizes (including 2.5 internodes, or approximately 3 cm in length) from these plants and grew them in 2.5‐inch pots within 32‐pot trays. As perennial *M. guttatus* often reproduces clonally in the native and invasive range (Truscott et al. 2006; van Kleunen 2007; Pantoja et al. 2018), clones permit performance measurements of the same genotypes across a range of temperatures using a mode of reproduction critical to its proliferation in riparian habitats. Another advantage of using cuttings rather than seedlings is that they are less prone to maternal effects (reviewed in Roach and Wulff 1987). Prior to temperature treatments, we allowed each set of clones 2 weeks to establish roots. During this period, cuttings were kept within chambers set to 20°C day/15°C night and bottom‐watered daily with a nutrient solution (Saravitz et al. 2009). We randomized cuttings both within and among chambers twice per week to reduce location effects. We subsequently transferred clones into a growth chamber programmed to one of 6 day/night temperature regimes (°C): 10/0, 20/10, 25/15, 30/20, 40/30, and 45/35. Over the course of the experiment, we randomly assigned these temperature treatments to one of three identical Percival LT‐105X chambers (Percival Scientific, Inc., Perry, IA), resulting in two full rounds of chamber use. For each temperature regime, we took two replicates (clones) of each genotype and randomized them among six trays. We subjected clones to each temperature regime with 16‐hour days and 8‐hour nights, according to procedures previously implemented by Paul et al. (2011), Sheth and Angert (2014), and Wooliver et al. (2020). Temperature regimes were replicated once, along with an additional replicate of the 45°C/35°C regime. Of the 1302 cuttings we took, 25 failed to establish, resulting in 1277 plants from 18 native and 13 invasive populations, representing 54 and 41 genotypes, respectively. We exposed clones to a given temperature regime for 1 week, during which trays were sub‐irrigated daily with water to prevent different rates of nutrient uptake at different temperatures.

Because perennial *M. guttatus* commonly reproduces vegetatively using stolons, stem and branch growth is a critical component of this species’ reproductive capacity (Truscott et al. 2006; van Kleunen 2007; Pantoja et al. 2018). Therefore, we measured relative change in stem length over the 7‐day temperature treatment. We chose this short treatment period because previous work with *M. guttatus* has documented significant growth responses to temperature in 1 week (Sheth and Angert 2014). Because high survival and growth of vegetative fragments following flood pulses have promoted spread throughout the U.K. range, short‐term, rapid growth of clones is an important performance metric for *M. guttatus* invasion success (Truscott et al. 2006). We quantified the length of the primary stem and the total length of branches (including stolons and secondary stems) before and after each temperature treatment (stem_in_ and stem_out_, respectively). Total length of branches was estimated as the number of branches multiplied by the length of an average branch approximately halfway down the primary stem. We then calculated relative growth rate (RGR) as the change in total stem length per initial stem length per day (Equation 1). Negative RGR values arising when clones lost stem or branch length due to dieback at temperature extremes were set to zero, and we excluded negative RGR values resulting from accidental damage (26 cuttings), resulting in a final dataset of 1251 individuals. Although RGR is not a measure of lifetime fitness, we used this metric as a proxy for fitness because size is related to reproductive output in other *Mimulus* species (Sheth and Angert 2018). Several studies have shown rapid growth responses to temperature over a short, 7‐day period in *M. guttatus* and other *Mimulus* species (Angert et al. 2011; Sheth and Angert 2014; Wooliver et al. 2020). We thus focus on RGR over this short time frame because (1) it represents a key functional trait that should influence survival and reproduction in this perennial species that commonly undergoes clonal reproduction; (2) it is a performance metric that is known to show rapid responses to temperature in this study system; and (3) it can be feasibly and easily measured on hundreds of individuals on a single day.

(1) $$\mathrm{RGR}=\frac{\left(\mathrm{ste}m_{\mathrm{out}}-\mathrm{ste}m_{\mathrm{in}}\right)}{\left(\mathrm{ste}m_{\mathrm{in}}\times \mathrm{number}\;\mathrm{of}\;\mathrm{days}\right)}.$$

### BAYESIAN TPC MODELS

We performed all analyses in R version 3.6.2 (R Core Team 2019). To generate TPCs for native and invasive populations of *M. guttatus*, we used a hierarchical Bayesian model (R package performr version 0.2; Tittes et al. 2019) to simultaneously fit curves predicting RGR across temperature for all populations while estimating uncertainty. As this model does not allow inclusion of random effects, we first averaged RGR across clones of each genotype at each temperature. This model also required scaling RGR values by the mean RGR across all data and centering daytime temperature around zero. Thus, we rescaled model outputs to reflect actual RGR values and temperatures. To improve the effectiveness of posterior sampling, we altered the default settings of the model to include a total of 4000 iterations per chain (with the default 50% warmup) and a maximum tree depth of 15 (Gelman et al. 2014), resulting in 8000 posterior draws. These settings increased model convergence (indicated by the potential scale reduction factor, *Ȓ*, equaling 1 for all parameters) and reliability of posterior sampling (effective samples were well over 1000 for population‐level parameters; Gelman et al. 2014). This model uses a derivation of Kumaraswamy's probability density function to fit performance curves across a continuous environmental gradient. We assessed the fit of Bayesian models using a posterior predictive *P*‐value, which uses a test variable to give the probability that values drawn from the simulated posterior predictive distribution will exceed the observed values. *P*‐values closest to 0.5 indicate adequate fit between the modeled and observed data (Gelman et al. 2014). The Bayesian *P*‐value for the overall model was 0.53, and *P*‐values for each population ranged from 0.2 to 0.82 (Table S2).

From these models, we obtained estimates of thermal optimum (*T*_opt_) and thermal performance breadth (*T*_breadth_) for each population (Table S3). We selected critical values for *T*_breadth_ from 100 equally spaced points along the temperature axis closest to the temperatures corresponding to 50% of maximum performance. Although these models also generated estimates of critical upper and critical lower thermal limits (temperatures at which RGR decreases to zero), these estimates extended beyond our temperature treatments. We thus excluded these parameters from our analyses.

### STATISTICAL ANALYSES FOR HYPOTHESIS TESTING

We tested for divergence in TPC parameters between native and invasive populations of *M. guttatus* independent of latitude by conducting post hoc comparisons of TPC model iterations. For each iteration, we calculated the average TPC parameter of interest among populations of the native and invasive ranges and subtracted the invasive average from the native average to obtain a pairwise difference. As such, a positive pairwise difference would indicate that the native range has a higher among‐population average than the invasive range, and vice versa. Then, we calculated the mean and 95% credible interval of the pairwise difference in each TPC parameter between the native and invasive range. We interpreted a statistically significant difference if the 95% credible interval did not include zero.

To determine whether TPCs of invasive populations have rapidly adapted to temperature and latitudinal gradients in the novel range, we compared latitudinal and thermal clines of TPC parameters in the native and invasive range using general linear models. To test whether *T*_opt_ decreased with latitude or increased with mean annual temperature, we modeled *T*_opt_ as a function of either latitude or mean annual temperature, range, and their interaction. Similarly, to evaluate whether *T*_breadth_ increased with latitude in the native range, decreased with latitude in the invasive range, and increased with temperature seasonality in both ranges, we modeled *T*_breadth_ as a function of latitude or temperature seasonality, range, and their interaction. We focused on mean annual temperature and annual temperature seasonality (calculated as the standard deviation of mean monthly temperature), rather than temperature variables restricted to a subset of the year because of the long growing season for perennial populations of *M. guttatus* in both the native (Hall and Willis 2006) and invasive ranges. Specifically, coastal populations of *M. guttatus* can flower from March through October, and seeds can germinate in autumn and overwinter as small rosettes (Hall and Willis 2006; Sheth and Vallejo‐Marin, pers. obs.). These perennial populations typically live near permanent water sources and remain active even during the warmest, driest months of the year (Lowry and Willis 2010). Thus, annual temperature variables capture the biologically relevant times of the year when *M. guttatus* is active better than seasonal variables. Climate data were obtained from WorldClim version 2 (∼1‐km resolution, 1970–2000, Fick and Hijmans 2017). We used AIC to compare models with and without the interaction term to account for the possibility of clines of similar magnitude and direction in both ranges. A significant interaction term would indicate divergent evolution of clines between ranges. In contrast, a significant effect of latitude or temperature, along with a nonsignificant or absent interaction term, would indicate parallel evolution of clines across ranges, although a nonsignificant interaction term could also arise due to a lack of statistical power.

## Results

### OVERALL SHIFTS IN TPC PARAMETERS BETWEEN THE NATIVE AND INVASIVE RANGES

**Figure 3:**
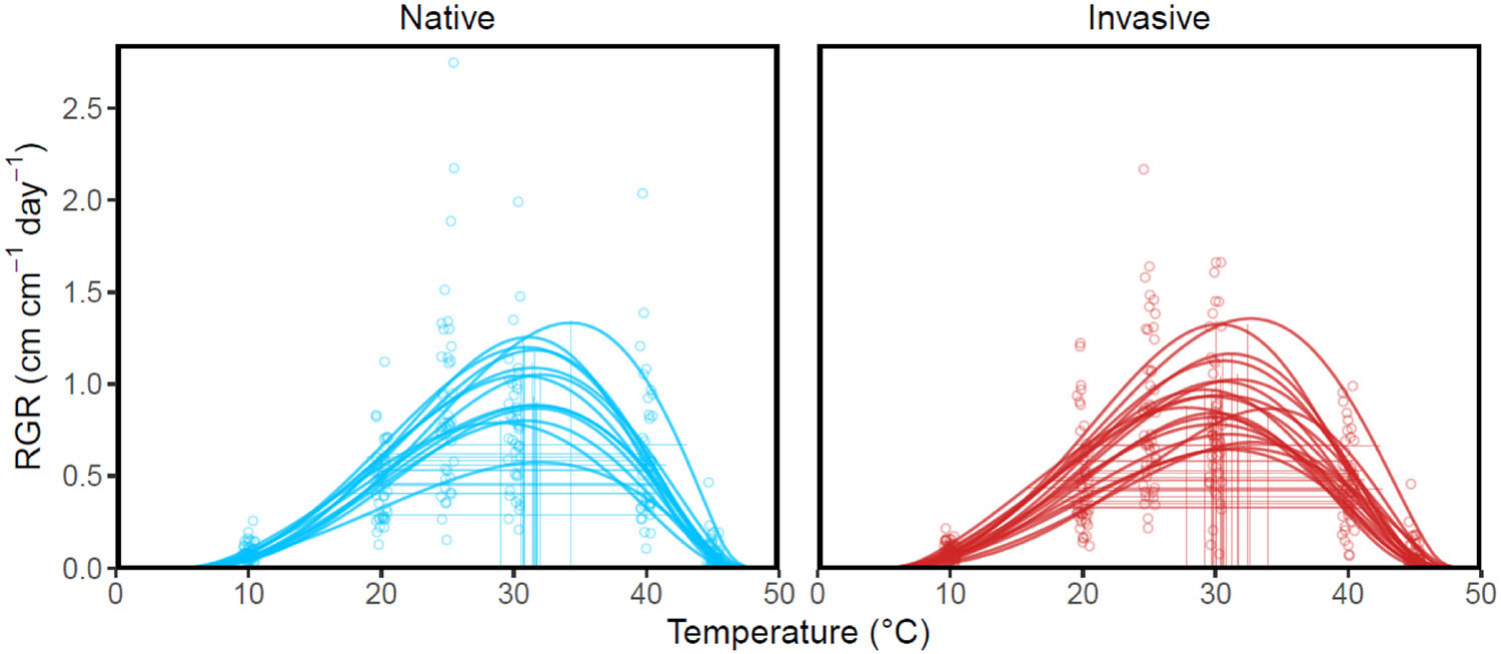
Thermal performance curves of 18 native and 13 invasive populations of *M. guttatus*. Vertical lines represent thermal optima (*T*_opt_) and horizontal lines denote thermal performance breadth (*T*_breadth_). Points represent genotype‐level mean relative growth rate (RGR) at a given daytime temperature.

We found no support for the hypothesis that *M. guttatus* has evolved within its invasive range via TPC shifts. Pairwise comparisons of TPC parameters between ranges revealed that *T*_opt_ and *T*_breadth_ exhibited 95% credible intervals that included zero (pairwise difference for *T*_opt_ = −0.654°C, 95% CI = −1.405, 0.108; pairwise difference for *T*_breadth_ = 0.018°C, 95% CI = −1.191, 1.239), indicating that neither differed significantly between ranges (Figures 3 and S2).

### CLINES OF TPC PARAMETERS

**Figure 4:**
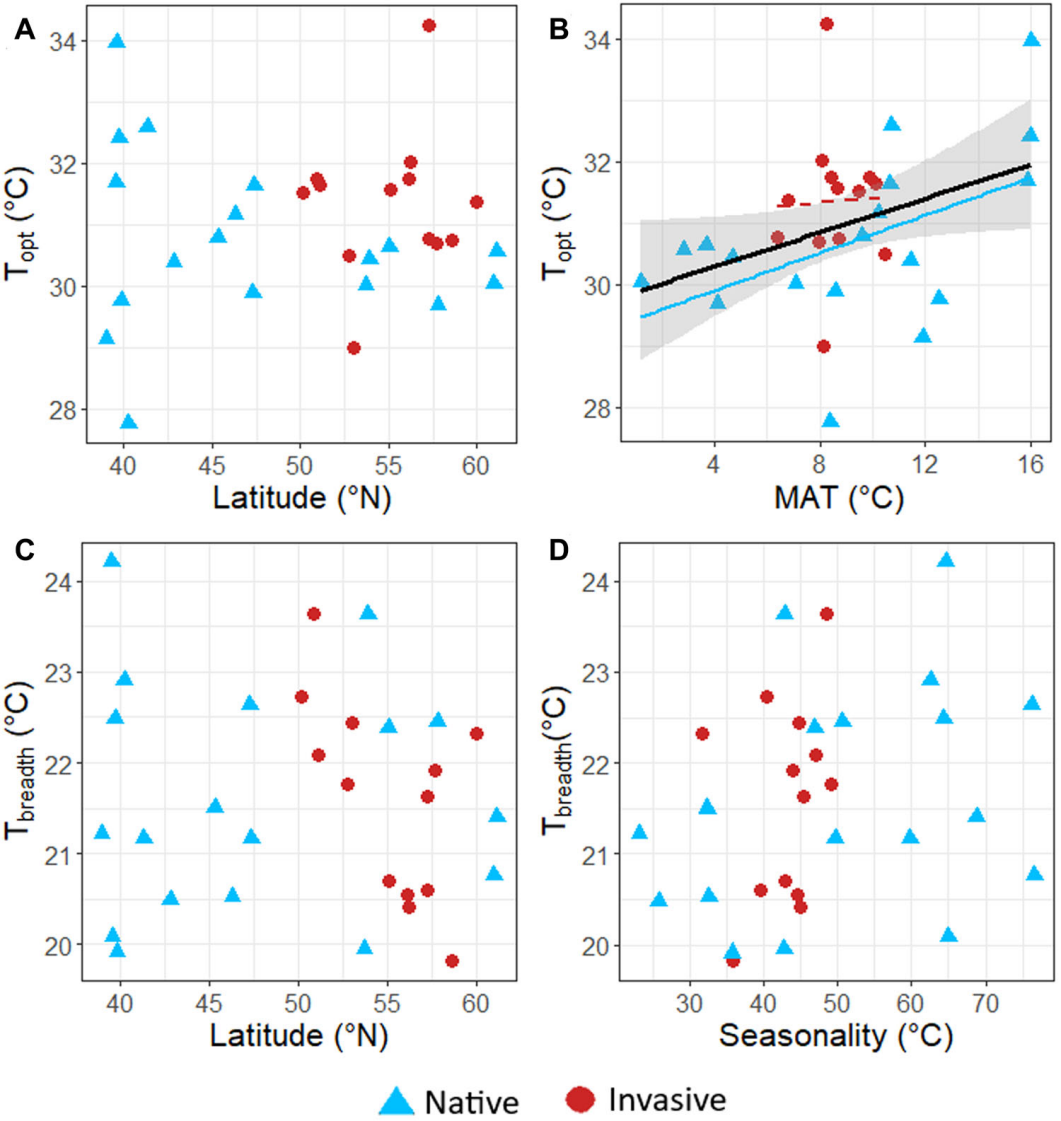
Relationships between population‐level thermal optimum (*T*_opt_) or thermal performance breadth (*T*_breadth_) and latitude, mean annual temperature (MAT), or temperature seasonality. A main effect regression line (black) is shown to indicate an overall cline of MAT (α = 0.05; Table 1) with *T*_opt_. However, this cline was driven by significant adaptive differentiation within the native range (solid blue line; *P* < 0.05), rather than the invasive range (dashed red line; *P* > 0.05).

Overall, latitudinal and thermal clines varied in strength depending on the TPC parameter and/or the geographic range. The final models (with lowest AIC) for *T*_opt_ as a function of latitude or mean annual temperature (MAT) did not include an interaction between latitude or MAT and range (Table 1). Failing to support our hypothesis, *T*_opt_ did not decrease with latitude (Table 1; Figure 4A). Further, there was no main effect of range when assessing clines of *T*_opt_ across latitude (Table 1). *T*_opt_ increased with MAT, but there was also no main effect of range on *T*_opt_ when assessing clines across MAT (Table 1; Figure 4B). To further dissect the relatively low variance explained by this model (Adj. *R* ^2^ = 0.155), we conducted range‐level models. We found that the cline of *T*_opt_ increasing with MAT in our full model was primarily driven by adaptive differentiation within the native range (β = 0.153, *P =* 0.036, Adj. *R* ^2^ = 0.199), whereas there was no relationship between MAT and *T*_opt_ in the invasive range (β = 0.038, *P =* 0.899, Adj. *R* ^2^ = −0.089).

**Table 1:** Regression coefficients and *P*‐values from full general linear models relating response variables (*T*_opt_: thermal optimum; *T*_breadth_: thermal performance breadth) to predictors

Full Model						
Response	**Predictor (β1, β2, β3)**	**β1, *P***	**β2, *P***	**β3, *P***	**AIC**	**Adj. *R* ^2^**
*T*_opt_	Lat, R, Lat × R	0.045, *P* = 0.712	3.812, *P* = 0.590	–0.087, *P* = 0.504	111.848	–0.002
	**Lat, R**	**–0.033, *P* = 0.409**	**–0.908, *P* = 0.123**	**–**	**110.372**	**0.018**
	MAT, R, MAT × R	0.038, *P* = 0.899	–1.738, *P* = 0.521	0.115, *P* = 0.710	107.545	0.128
	**MAT, R**	**0.148, *P =* 0.028** *	**–0.748, *P* = 0.108**	**–**	**105.708**	**0.155**
*T*_breadth_	**Lat, R, Lat × R**	**–0.201, *P* = 0.069**	**–11.209, *P* = 0.077**	**0.204, *P* = 0.079**	**103.385**	**0.020**
	Lat, R	–0.018, *P =* 0.623	–0.120, *P =* 0.820	–	104.993	–0.062
	TS, R, TS × R	0.058, *P* = 0.407	1.265, *P* = 0.689	–0.034, *P* = 0.640	104.236	–0.008
	**TS, R**	**0.026, *P =* 0.117**	**–0.197, *P =* 0.662**	**–**	**102.492**	**0.020**

*Note*: Here, Lat = Latitude and R = Range. β1 indicates the main effect of either latitude, mean annual temperature (MAT), or temperature seasonality (TS). β2 indicates the main effect of range (invasive vs. native). β3 indicates the interaction between range and the predictor corresponding to β1. Based on AIC, we chose bolded models as the final model for each respective cline.

* *P* < 0.05; **P < 0.01; ***P < 0.001.

The final model (with lowest AIC) of *T*_breadth_ as a function of latitude included an interaction between latitude and range (Table 1). Contrary to the prediction that *T*_breadth_ would increase with latitude in the native range and decrease with latitude in the invasive range, *T*_breadth_ showed no relationship with latitude (Table 1; Figure 4C). There was not a significant main effect of range on *T*_breadth_, and the relationship between latitude and *T*_breadth_ did not differ between ranges (Table 1). The final model (with lowest AIC) of *T*_breadth_ as a function of temperature seasonality did not include an interaction term (Table 1). We found no cline of *T*_breadth_ with temperature seasonality (Table 1; Figure 4D), failing to support the climate variability hypothesis. Further, there was no main effect of range on *T*_breadth_ when assessing clines of *T*_breadth_ across temperature seasonality (Table 1).

## Discussion

We compared TPCs of 18 native and 13 invasive populations of *M. guttatus* to test key hypotheses about the role of climatic niche evolution in facilitating biological invasions. Our results provided no support for the hypothesis that thermal optimum and breadth vary between the native and invasive ranges (Figure S2). Further, contrary to the hypothesis that the invasive range will re‐establish phenotypic clines found in the native range, there was limited evidence for the re‐establishment of clines in TPC parameters (Table 1; Figure 4). Rather than supporting the hypothesis that TPC evolution has played a role in facilitating *M. guttatus* invasion in the United Kingdom, these results provide physiological support for thermal niche conservatism in the invasive range. Below, we discuss the implications of these findings in light of the evolutionary processes that could contribute to biological invasion.

### EVOLUTION OF TPCs

We found no support for the hypothesis that invasive populations have undergone changes in mean TPC parameters relative to native populations (Figures 3 and S2). Instead, *T*_opt_ and *T*_breadth_ were similar between native and invasive ranges, consistent with the finding of climatic niche conservatism between the native North American and invasive European (including the United Kingdom and continental Europe) ranges of *M. guttatus* based on niche models (Da Re et al. 2020). However, like many other invasive species (Liu et al. 2020), *M. guttatus* has undergone substantial niche unfilling (Da Re et al. 2020), where invasive European populations are found in a comparatively small subset of the climatic conditions (including temperature; Figure S1) occupied in the native range. Although the range of temperatures is narrower in the introduced range (Figure 2C), *T*_breadth_ did not differ between ranges (Figure S2), suggesting that invasive *M. guttatus* populations are poised to occupy greater climatic niche space should it become available.

Insufficient standing genetic variation could contribute to the evolutionary stasis of TPC parameters in the invasive range (Dlugosch et al. 2015; Prentis et al. 2008), but the highly admixed nature of invasive *M. guttatus* populations has allowed for maintenance of relatively high genetic diversity (Puzey and Vallejo‐Marín 2014; Pantoja et al. 2017; Vallejo‐Marín et al. 2021). These high levels of genetic variation likely contributed to the maintenance of broad TPCs and allowed these populations to easily tolerate the narrower range of temperatures in the invasive U.K. range. In sum, the invasive populations have yet to undergo significant divergence in TPCs from native populations due to overlap in thermal niche space, and therefore a lack of selective pressure, rather than insufficient genetic variation. Maintenance of broad thermal tolerance and high genetic admixture in the invasive range could allow *M. guttatus* populations to withstand rapid changes in temperatures, increasing risks that invasive populations will proliferate under climate change.

### EVOLUTION OF CLINES

Although temperature varies predictably with latitude in both the native and invasive range of *M. guttatus* (Figure 2C,D), and there is ample evidence that rapid adaptation can facilitate invasion success (Oduor et al. 2016), our results did not support the hypothesis of re‐established clines in the invasive range. Mean annual temperature was positively related to *T*_opt_ in the native range, but not in the invasive range (see *Results*; Figure 4B). The absence of this cline in the invasive range implies weaker selection on *T*_opt_ or insufficient time for adaptation in the invasive range. Given that invasive populations of *M. guttatus* have maintained similar *T*_breadth_ as native populations, selection in the invasive range with a relatively narrow range of mean annual temperatures would not likely favor adaptive differentiation in *T*_opt_ (Figure 2C).

Failing to support the climate variability hypothesis (Dobzhansky 1950; Janzen 1967; Stevens 1989), *T*_breadth_ was not related to latitude or temperature seasonality in either range. There are many possible explanations for a lack of clines in *T*_breadth_ in native populations. First, the weak relationship between temperature seasonality and latitude in the native range (Figure 2B) makes it particularly unlikely for a latitudinal cline in *T*_breadth_ to evolve. Second, microclimatic variation could cause populations of *M. guttatus* to occupy areas with either highly similar or drastically different regimes of temperature seasonality, which could hinder the detection of clines at broader macroclimatic scales (Franco and Nobel 2003; Rashkovetsky et al. 2006; De Frenne et al. 2013). Although additional analyses of variation in TPC parameters within populations would be helpful for dissecting this hypothesis, our model was unable to fit genotype‐level curves due to insufficient replication. Finally, high gene flow among native populations could swamp adaptation of TPCs to local thermal regimes (Paul et al. 2011). Rather than evolving thermal performance breadth in response to seasonality, native populations may consist of general‐purpose genotypes that contributed to their successful establishment as an invasive species (van Kleunen et al. 2011). This conclusion is consistent with the finding of high rates of gene flow among coastal populations occupying similar latitudes in the native range relative to our study populations (Twyford and Friedman 2015). Nonetheless, our findings are particularly surprising given the re‐establishment of phenotypic clines in the introduced ranges of several species (Hernández et al. 2019; van Boheemen et al. 2019; McGoey et al. 2020). Overall, our results suggest that although native *M. guttatus* populations are adaptively differentiated by thermal optima, populations are equally tolerant across a broad temperature gradient in western North America. Such homogenization of *T*_breadth_ among native populations may have allowed *M. guttatus* to maintain a broad thermal niche space upon introduction.

### CAVEATS

One caveat of our study is that we focused on one performance metric, stem and branch RGR over a week‐long period, but to fully understand performance trade‐offs and variation in performance across temperatures, multiple performance metrics that capture varying degrees of investment to above and belowground growth over longer experimental periods and across multiple life stages are necessary. Ultimately, performance metrics that approximate lifetime fitness could yield stronger phenotypic clines. Nevertheless, short‐term RGR of cuttings captures a performance metric of perennial *M. guttatus* that affects traits associated with fitness, such as size, nutrient uptake, and competitive ability, and ultimately influences achievable vegetative reproduction, numbers of flowers, and seed production (Lambers and Poorter 1992; Pugnaire and Valladares 2007). Second, given that invasive U.K. populations of *M. guttatus* do not occupy dramatically distinct climatic niche space relative to native populations, our ability to detect rapid climatic niche evolution in the invasive range was necessarily limited. However, the detailed characterization of invasive U.K. populations and their central role in facilitating other invasions worldwide meant that characterizing potential rapid evolutionary shifts in thermal performance was an essential first step to study its evolution elsewhere. Future TPC comparisons that include introduced populations from additional regions would further contribute to our understanding of the role of thermal niche evolution in the global range expansion of *M. guttatus*. Although other studies hint that climatic adaptation during invasions may not be widespread (Eyster and Wolkovich 2021), further work exploring these ideas in well‐characterized systems where there is strong temperature variation between the native and invasive ranges is needed. Third, we focused on temperature, but evolution along other niche axes such as precipitation and edaphic properties could also contribute to invasion success (Hall and Willis 2006; van Kleunen and Fischer 2008). Finally, the biotic environment may also differ in the introduced range (Holeski et al. 2013; Rotter et al. 2019; Thawley et al. 2018), implying that many factors beyond abiotic conditions can play a part in adaptation to novel conditions during invasions.

## Conclusions

Plant invasions have been widely studied for land management, but little consensus exists on the ecological and evolutionary processes that facilitate invasion success. Similar thermal optima and breadth in the invasive and native ranges of *M. guttatus* and the absence of a cline in thermal breadth even in the native range suggest that the consistently broad thermal tolerance of native populations, rather than rapid TPC evolution, may have contributed toward successful establishment in the invasive range. These results provide physiological support for previous findings of niche conservatism between native and invasive *M. guttatus* (Da Re et al. 2020) and bolster the value of the climatic niche models based on native occurrences to predict potential invasions in this species. Comparisons of the evolution of thermal performance across broad environmental gradients in a species’ native and invasive range can inform predictions of which species will become aggressive invaders and help us understand how they may fare under rapid climate change. Species that are genetically predisposed toward a broad thermal tolerance may rapidly expand in their invasive range as climatic conditions shift. These predictions will be invaluable in preparing for future species invasions, strengthening our efforts to manage invasive species in the face of rapid climate change.

## AUTHOR CONTRIBUTIONS

AQ and SNS conceived of and designed the study with feedback from MVM. AQ collected the data. AQ and RW analyzed the data. AQ and SNS led the writing, with substantial contributions from RW and MVM. All authors contributed critically to the drafts and gave final approval for publication.

## DATA ARCHIVING

Data are available at doi:10.5061/dryad.7wm37pvvq, and all data and scripts associated with this study are available at https://github.com/akquerns/Evolution‐Letters‐2022‐Guttatus‐TPC.

Associate Editor: Zachariah Gompert

## Supporting information


**Table S1**. Seed collection sites for potential *M. guttatus* populations to be included in thermal performance experiments.
**Table S2**. Posterior predictive *P*‐values generated for each *M. guttatus* population's thermal performance curve.
**Table S3**. Mean thermal performance curve parameter values for each population, and 95% credible intervals for these parameter values, generated using a Bayesian model.
**Figure S1**. Results of PCA exploring variation in 11 temperature variables from WorldClim v. 2.0 across all populations considered for use in our study (Supplementary methods, Table S1; 1970‐2000, Fick & Hijmans 2017).
**Figure S2**. Pairwise comparisons of range‐level mean thermal performance curve parameters. Parameters shown are (A) *T_opt_* (thermal optima) and (B) *T_breadth_* (thermal performance breadth).
